# Ten Years in Remission: The Long-Term Journey of a Breast Cancer Survivor

**DOI:** 10.7759/cureus.98291

**Published:** 2025-12-02

**Authors:** Sofia Peixoto, Raquel Basto, Enrique Dias

**Affiliations:** 1 Department of Medical Oncology, Unidade Local de Saúde de Gaia e Espinho, E.P.E. (ULS Gaia-Espinho), Vila Nova de Gaia, PRT

**Keywords:** her2+, long-term survival, metastatic breast cancer, palliative chemotherapy, personalized treatment, remission

## Abstract

Metastatic breast cancer (MBC) remains a challenging clinical entity, with long-term remission being rare. We report the case of a 51-year-old woman diagnosed in 2009 with HER2-positive, hormone receptor-positive invasive carcinoma of the right breast with extensive hepatic metastases. Following multimodal treatment, including surgery, multiple lines of chemotherapy, targeted therapy, and hormonal therapy, she achieved complete remission of metastatic disease in 2016 and remained disease-free until her death in 2025. Her clinical course was later complicated by the development of a second primary gastric adenocarcinoma in 2024, as well as treatment-related hepatic toxicity. This case illustrates that, in selected patients, prolonged remission can be achieved through a multidisciplinary, personalized treatment strategy and personalized approach. At the same time, it underscores the critical importance of long-term surveillance for secondary malignancies and late treatment-related adverse effects.

## Introduction

Metastatic breast cancer (MBC) remains a major clinical challenge, with a global median survival of 18-24 months [1]. Advances in targeted therapies, particularly for HER2-positive (HER2+) subtypes, have transformed treatment paradigms, enabling durable responses and, in rare instances, prolonged remissions [1,2]. Studies suggest that up to 3% of cases may achieve long-term cure, particularly in younger individuals with oligometastatic disease who undergo aggressive multimodal treatment [2]. Survival beyond 10 years has been associated with factors such as hormone receptor positivity (ER+/PR+), HER2 positivity, absence of visceral metastases, and sustained responses to anti-HER2 therapies [2,3]. Nevertheless, long-lasting remission in MBC with extensive hepatic involvement remains exceptional.

The literature indicates that durable disease control may be facilitated by prolonged benefit from combined regimens (e.g., lapatinib with endocrine therapy), local treatment of oligometastases, and molecular stability (such as absence of PI3K or *ESR1* mutations) [2,3]. However, discontinuation of therapy in such contexts, whether due to cumulative toxicity or comorbidities, remains insufficiently studied, creating a therapeutic dilemma [2].

This case describes a patient with HER2+ MBC and multinodular hepatic metastasis who achieved and maintained clinical remission for 15 years under continuous palliative chemotherapy, challenging the traditional expectation of inevitable progression in visceral disease. The later development of a second primary gastric adenocarcinoma, in the absence of breast cancer recurrence, underscores the importance of multidisciplinary surveillance in long-term survivors, as well as dedicated follow-up programs tailored to monitor both late treatment effects and secondary malignancies.

The patient's outcome illustrates the value of a multidisciplinary strategy that integrates personalized systemic therapy with proactive management of adverse events, highlighting the interplay between pharmacological advances and individual resilience. Overall, this case supports the hypothesis that, although rare, a functional cure of MBC is attainable in selected subgroups when aggressive local measures, targeted therapies, and ongoing adaptation to tumor biology are combined [3].

## Case presentation

**Figure 1:**
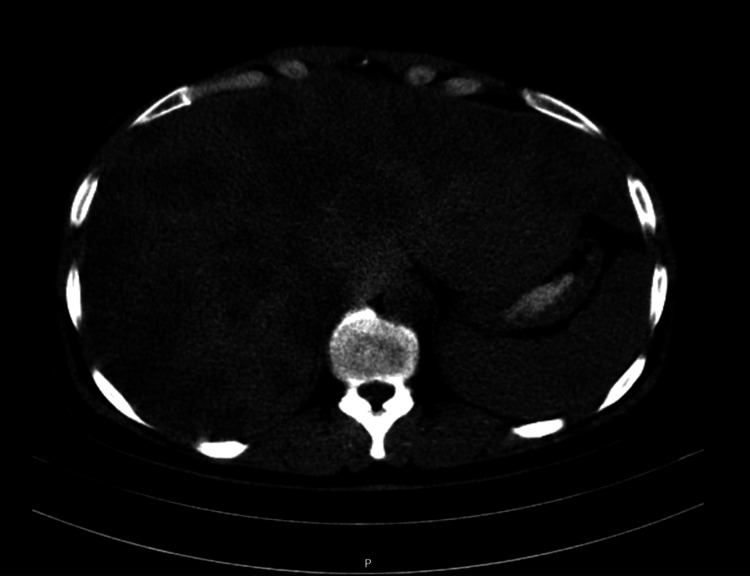
Axial CT image at diagnosis (2009) showing extensive multinodular hepatic metastases.

A 51-year-old woman, an active smoker (33 pack-years), with a history of depressive syndrome and no relevant family history, was referred to the oncology clinic in 2009 for evaluation of an infiltrative right breast lesion measuring approximately 8 x 9 cm associated with right axillary adenopathy. Core biopsy confirmed invasive breast carcinoma (ER+, HER2+, cErB2 overexpression). Staging CT (thoraco-abdominal-pelvic) revealed extensive multinodular hepatic metastasis, consistent with stage cT4N+M1 (American Joint Committee on Cancer (AJCC) 7th edition) (Figure 1).

She received six cycles of palliative chemotherapy with 5-fluorouracil (500 mg/m²), epirubicin (75-100 mg/m²), and cyclophosphamide (500 mg/m²) (FEC), administered intravenously every 21 days, achieving marked regression of the primary tumor and hepatic metastases. In January 2010, she underwent a modified radical mastectomy of the right breast, performed for local disease control and patient comfort. The pathology revealed necrosis without any residual invasive carcinoma, multiple foci of high-grade intraductal carcinoma with microinvasion, and metastases in four axillary lymph nodes. This was followed by six cycles of docetaxel (75 mg/m² IV, every 21 days) and trastuzumab (8 mg/kg IV loading dose, then 6 mg/kg IV every 21 days). Trastuzumab was discontinued in June 2011 due to cardiotoxicity, evidenced by a >10% decline in left ventricular ejection fraction (LVEF) on echocardiography. The patient was subsequently re-evaluated, but follow-up echocardiography showed persistent LVEF reduction; therefore, trastuzumab was not reintroduced. She was subsequently maintained on hormonal therapy with tamoxifen (20 mg orally once daily).

**Figure 2:**
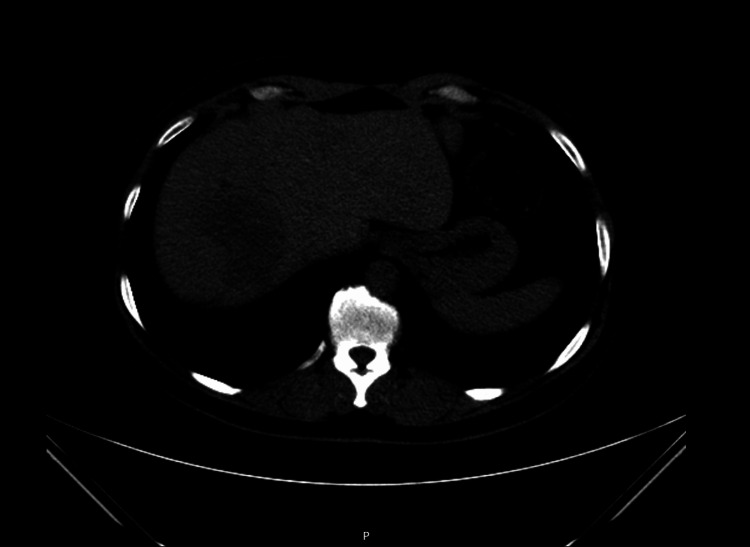
Axial CT image in 2015 showing recurrent hepatic metastases.

**Figure 3:**
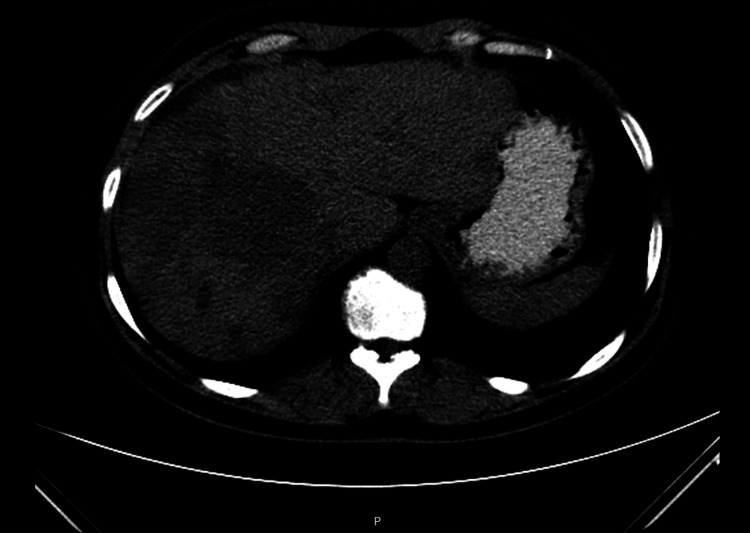
Axial CT image in March 2016 showing diffuse hepatic progression.

She remained progression-free on tamoxifen for approximately four years, with no relevant toxicity and with good tolerance. In August 2015, new hepatic metastases were detected (Figure 2). A regimen of lapatinib (1,500 mg orally once daily) and exemestane (25 mg orally once daily) was initiated but discontinued in March 2016 after disease progression with diffuse liver involvement and ascites (Figure 3). She then received ado-trastuzumab emtansine (T-DM1, 3.6 mg/kg every 21 days). This treatment was discontinued after evidence of hepatic disease progression. In July 2016, she commenced lapatinib (1,250 mg daily, continuously) plus capecitabine (2,000 mg/m²/day, in two divided doses on days 1-14 of a 21-day cycle), which led to complete remission of the hepatic metastases, as confirmed on follow-up thoracoabdominal-pelvic CT, with good clinical tolerance.

Complications related to lapatinib and capecitabine developed three years after treatment initiation, including portal hypertension, hepatic cirrhosis secondary to chemotherapy, and an episode of upper gastrointestinal bleeding. These were managed conservatively and did not necessitate discontinuation of oncological therapy.

**Figure 4:**
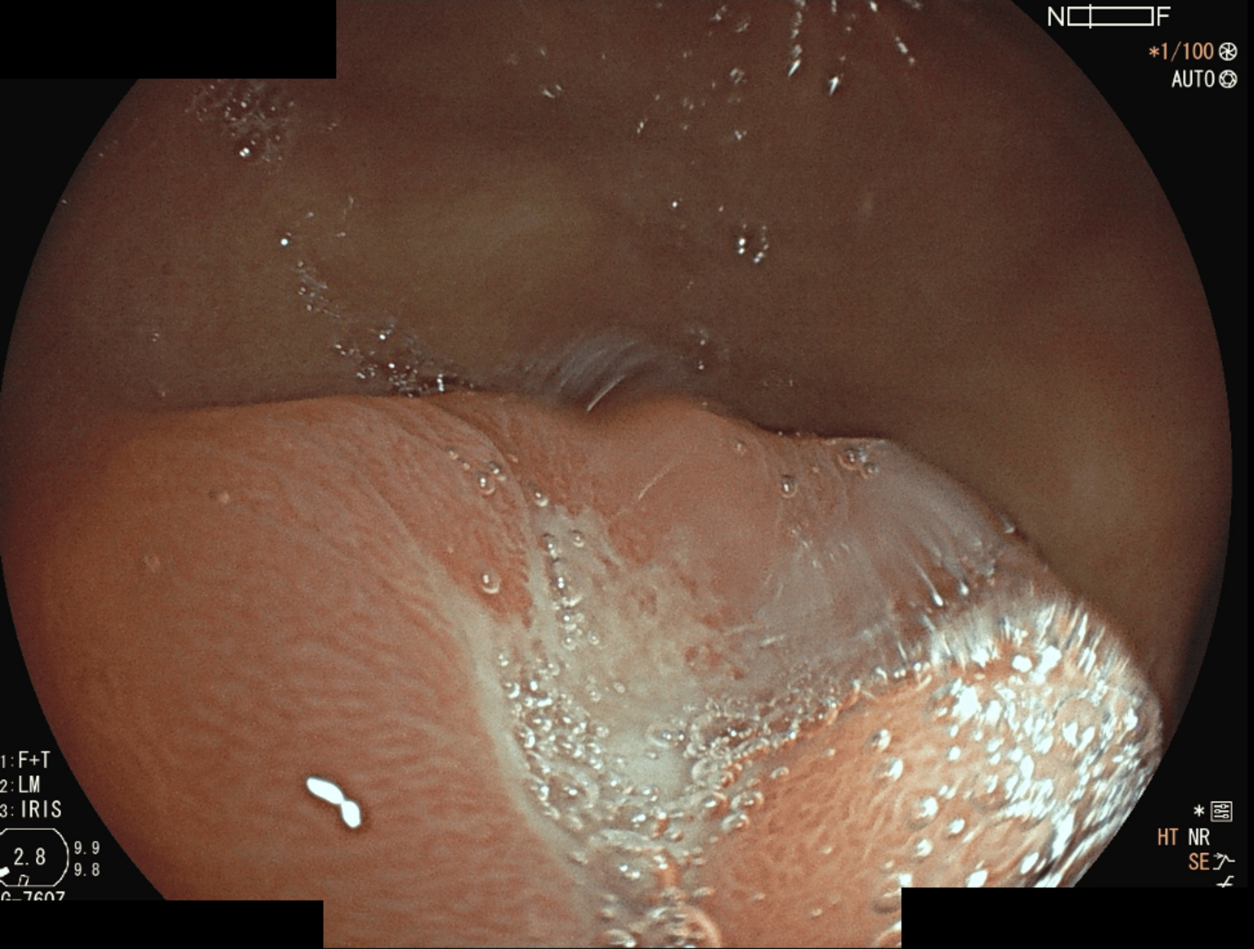
Upper gastrointestinal endoscopy performed in July 2024, showing a gastric ulcer later confirmed as moderately differentiated tubular adenocarcinoma.

In July 2024, a routine upper endoscopy revealed a gastric ulcer (Figure 4), with pathology confirming a moderately differentiated tubular gastric adenocarcinoma (uT1bN0M0, AJCC 8th edition). Following multidisciplinary discussion, lapatinib and capecitabine were suspended, and the patient underwent subtotal gastrectomy in November 2024. Postoperatively, she developed refractory ascites, spontaneous bacterial peritonitis, and portal vein thrombosis.

By April 2025, she remained free from oncological recurrence but had progressive clinical decline, with dependency in activities of daily living due to refractory ascites, requiring repeated paracenteses and an indwelling intraperitoneal drain. Despite multidisciplinary follow-up by Internal Medicine and Palliative Care, she died in April 2025.

**Figure 5:**
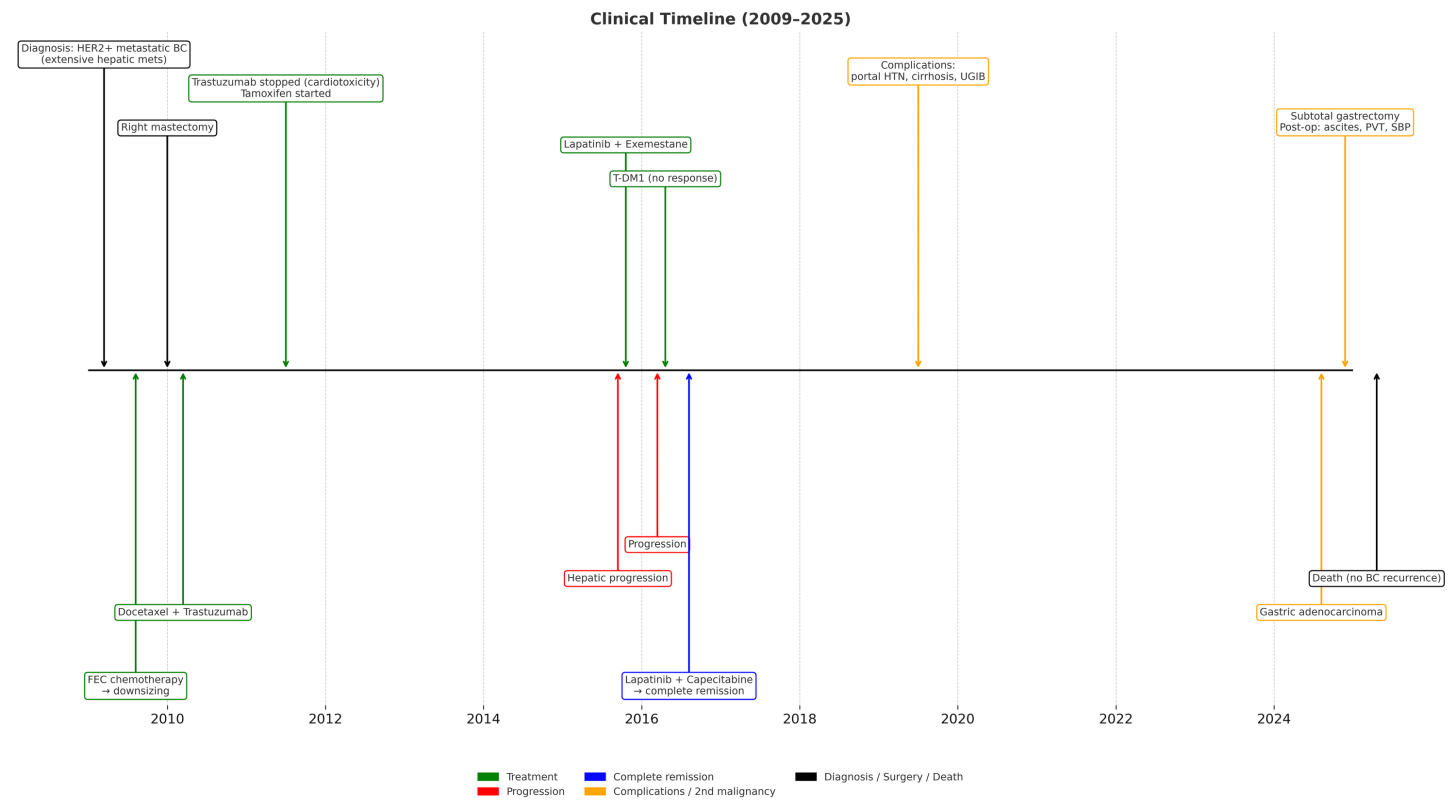
Clinical timeline (2009–2025). Summary of the oncological course of a 51-year-old woman with HER2-positive metastatic breast cancer and a second primary malignancy. Events are arranged chronologically along the central axis. Colors indicate treatment (green), disease progression (red), complete remission (blue), complications or second malignancy (orange), and neutral events (black). Abbreviations: UGIB: upper gastrointestinal bleeding; UGIE: upper gastrointestinal endoscopy; PVT: portal vein thrombosis; SBP: spontaneous bacterial peritonitis.

A timeline of her oncological course, major treatments, complications, and the development of a second malignancy over the 15-year follow-up is summarized in Figure 5.

## Discussion

The US National Cancer Institute defines an "exceptional responder" in MBC as a patient who demonstrates durable responses to therapies that are typically ineffective in more than 90% of cases [4]. Long-term survival is variably defined as ≥5 or ≥10 years post-diagnosis, depending on the study [3]. Favorable prognostic factors include younger age, premenopausal status, absence of comorbidities, and oligometastatic disease, particularly when confined to the lungs or lymph nodes [3,5]. In contrast, triple-negative breast cancer and the presence of brain or multiple visceral metastases are associated with poor outcomes [3,6].

Molecular subtype plays a central role: HER2-positive and hormone receptor-positive diseases correlate with improved long-term survival, often driven by sustained benefit from targeted therapies [3,7]. Reports suggest that long-term survivors frequently share characteristics such as HER2 positivity, endocrine sensitivity, and genomic stability, including the absence of PI3K mutations [3,7,8]. While triple-negative disease generally carries an unfavorable prognosis, rare cases of prolonged remission have been described following aggressive multimodal approaches [9].

The multidisciplinary approach, including the management of treatment-related adverse effects, is seen as a promising strategy for chronicization or cure in selected subgroups [3,5]. However, limitations persist due to the rarity of such cases and molecular heterogeneity [4,8].

## Conclusions

This case highlights the complexity of treating HER2-positive MBC and demonstrates that prolonged remission is achievable through personalized, multidisciplinary strategies. Although a cure remains rare, long-term disease control is possible, particularly when systemic therapy is combined with timely local interventions and vigilant surveillance.

As the number of long-term survivors increases, the establishment of dedicated survivorship clinics for this population is crucial. These clinics provide structured surveillance, facilitate early detection and management of late treatment effects, and deliver comprehensive support tailored to patients' evolving needs. Furthermore, they create valuable opportunities for research into survivorship care, ultimately contributing to the development of evidence-based best practices in this emerging field.
